# Supplementary material for: Content-rich biological network constructed by mining PubMed abstracts
Source: BMC Bioinformatics. 2004 Oct 8;5:147. doi: 10.1186/1471-2105-5-147 (PMC528731; doi:10.1186/1471-2105-5-147)
Supplement: Additional File 2 — The original results of the above study (non-essential files are deleted to keep the file size under the limit set by BMC bioinformatics). [file 1471-2105-5-147-S2.bz2 › chilibotAdditionalFile2/dip40/470ID9660793E10526/html/CCL7_CXCR3.html]

 


 **CCL7** and **CXCR3** 
  
Found 39 abstracts in PubMed, retrieved 39.  
 

 What does Google say? 
 PDF only 
| .edu only 

---

**Interactive relationship** (e.g. stimulation, inhibition, etc)

**Inhibitory relationship**- I TAC, Mig, and IP10  [ **CXCR3** ]  compete for the binding of eotaxin to CCR3 bearing cells and inhibit migration and Ca 2 changes induced in such cells by stimulation with eotaxin, eotaxin 2, MCP 2 monocyte chemottractant protein 2, MCP 3  [ **CCL7** ] , MCP 4, and RANTES regulated on activation normal T cell expressed and secreted.  Ref: 11110785 J Biol Chem, 2001
**Stimulatory relationship**- At 26 weeks postirradiation, messages encoding the chemokines BLC now known as Scyb13, C10 now known as Scya6  [ **CCL7** ] , IP 10  [ **CXCR3** ]  now known as Scyb10, MCP 1 now known as Scya2, MCP 3  [ **CCL7** ]  now known as Scya7  [ **CCL7** ] , MIP 1gamma now known as Scya9, and RANTES now known as Scya5 and the chemokine receptors Ccr1, Ccr2, Ccr5 and Ccr6 were elevated in fibrosis sensitive C57BL 6 mice.  Ref: 11839087 Radiat Res, 2002
- 1 LARC CCL20 whose mRNA level increased rapidly at 3 h post challenge and then gradually decreased, 2 JE CCL2, MARC  [ **CCL7** ]   **CCL7** , MIP 1gamma CCL9, monocyte chemoattractant protein MCP 5 CCL12, ELC CCL19 and BRAK CXCL14 whose mRNA levels increased with time and reached the maximum at 6 9 h post challenge, 3 LIX CXCL5, Mig CXCL9 and IP 10  [ **CXCR3** ]  CXCL10 whose mRNA levels increased gradually at least up to 12 h post challenge, and 4 SLC CCL21 whose mRNA level decreased gradually with time after challenge.  Ref: 12644322 Immunol Lett, 2003
**Neutral relationship**- Transfected cells expressing high levels of LESTR mRNA did not bind radiolabeled NPY, IL 8, NAP 2, GRO alpha, PF4, IP10  [ **CXCR3** ] , MCP 1, MCP 3  [ **CCL7** ] , MIP 1 alpha, HC14, I309, RANTES, C3a, or LTB4.  Ref: 8276799 J Biol Chem, 1994

**Non-interactive relationship** (e.g. studied together, co-existance, homology, etc.)

- Increased expression of IP 10  [ **CXCR3** ] , IL 8, MCP 1, and MCP 3  [ **CCL7** ]  in ulcerative colitis.  Ref: 10433925 Am J Pathol, 1999
- Expression of IL 8, MCP 1, MCP 3  [ **CCL7** ] , and IP 10  [ **CXCR3** ]  was significantly higher in pouchitis than normal pouch.  Ref: 12827410 Int J Colorectal Dis< MedlineTA>Int J Colorectal Dis, 1999
- Eosinophil chemotactic chemokines eotaxin, eotaxin 2, RANTES, monocyte chemoattractant protein 3  [ **CCL7** ]  MCP 3  [ **CCL7** ] , and MCP 4, and C C chemokine receptor 3  [ **CXCR3** ]  expression in bronchial biopsies from atopic and nonatopic Intrinsic asthmatics.  Ref: 10570327 J Immunol, 1999
- The percentage of cells expressing IP 10  [ **CXCR3** ] , IL 8, MCP 1, and MCP 3  [ **CCL7** ]  was significantly enhanced in all UC samples as compared to controls.  Ref: 10433925 Am J Pathol, 1999
- From among the 13 chemokines examined, MCP 3  [ **CCL7** ] , MCP 4, RANTES, and IP 10  [ **CXCR3** ]  were detected in the disc from the idiopathic scoliosis, and MCP 3  [ **CCL7** ] , MCP 4, RANTES, IP 10  [ **CXCR3** ] , MIG, and MGSA alpha were detected in the infected or herniated discs.  Ref: 12131709 Spine, 2002
- Some of these chemokines as well as RANTES, MCP 3  [ **CCL7** ] , MCP 5, and cytokine response gene 2 CRG 2 IFN inducible protein 10 IP 10  [ **CXCR3** ]  were associated with Th1, but not Th2, pancreatic infiltrates.  Ref: 10072490 J Immunol, 1999
- IFN alpha or IFN gamma were found to directly enhance MCP 1, MCP 3  [ **CCL7** ] , and IP 10  [ **CXCR3** ]  mRNA expression.  Ref: 11022002 Virology, 2000
- We have studied the expression of interferon gamma inducible protein 10 IP 10  [ **CXCR3** ] , interleukin 8 IL 8, monocyte chemoattractant protein MCP 1, MCP 3  [ **CCL7** ] , and macrophage inflammatory protein MIP 1alpha in UC patients and control individuals to assess the role of these chemokines in disease progression.  Ref: 10433925 Am J Pathol, 1999
- The expression of MCP 1, MCP 3  [ **CCL7** ]  and IP 10  [ **CXCR3** ]  were significantly lower after antibiotic treatment.  Ref: 12827410 Int J Colorectal Dis< MedlineTA>Int J Colorectal Dis, 1999
- Colonic biopsies were taken endoscopically from patients and controls, frozen immediately and subsequently stained for IP 10  [ **CXCR3** ] , IL 8, MCP 1, MCP 3  [ **CCL7** ] , and MIP 1alpha in serial sections.  Ref: 10433925 Am J Pathol, 1999
- The RNA was reverse transcribed, and the resultant cDNA was amplified by PCR using primer pairs specific to the CXC chemokines IL 8, MGSA alpha, IP 10  [ **CXCR3** ] , MIG, the CC chemokines MCP 1, MCP 2, MCP 3  [ **CCL7** ] , MCP 4, MIP 1alpha, MIP 3alpha, RANTES, STCP 1, the C chemokine lymphotactin, and the glyceraldehyde phosphate housekeeping gene.  Ref: 12131709 Spine, 2002
- Neutralization of virus induced IFN alpha beta resulted in downregulation of virus induced IP 10  [ **CXCR3** ] , MCP 1, and MCP 3  [ **CCL7** ]  mRNA expression.  Ref: 11022002 Virology, 2000
- We have developed a four step procedure that allows for the complete purification of active C C MCP 1, MCP 2, MCP 3  [ **CCL7** ] , RANTES, MIP 1alpha and MIP 1beta and C X C IL 8, GRO alpha, GRO beta, GRO gamma, GCP 2, ENA 78, IP 10  [ **CXCR3** ] , PF 4, and CTAPIII betaTG NAP 2 chemokines from bulk volumes of culture supernatant.  Ref: 8812648 Methods< MedlineTA>Methods, 2000
- Because most of these genes have been known to either inhibit angiogenesis Mig, IP 10  [ **CXCR3** ] , inhibit tumor growth Gjb2, MCP3  [ **CCL7** ] , kill tumor cells Granzyme A and C, or induce expression of antitumor gene IRF 7, they may become promising therapeutic gene candidates for a combination gene therapy approach to cancer treatment.  Ref: 11960315 Gene Ther, 2002
- Analyses using cDNA array and Northern blot indicated that the genes responsible for the therapeutic effect of electro IFN alpha gene therapy included IRF 7, Granzyme A, Granzyme C, Gjb2, Krt14, Mig, IP 10  [ **CXCR3** ]  and MCP3  [ **CCL7** ] .  Ref: 11960315 Gene Ther, 2002
- CKs fell into four categories a type 1 dominant gamma interferon inducible protein IP 10  [ **CXCR3** ] , monokine induced by INF gamma MIG, macrophage inflammatory protein 2 MIP 2, lipopolysaccharide induced chemokine LIX, rodent growth related oncogene homologue KP, macrophage inflammatory protein 1alpha MIP 1alpha and 1beta MIP 1beta, lymphotactin, b type 2 dominant eotaxin, monocyte chemotactic protein 2 MCP 2 and 3 MCP 3  [ **CCL7** ] , liver and activation regulated chemokine LARC, T cell activation protein 3 TCA 3, c type 1 and type 2 co dominant MCP 1, MCP 5, monocyte derived chemokine MDC, thymus and activation related chemokine TARC, C10, and d constitutive lungkine, secondary lymphoid tissue chemokine SLC, EBI1 ligand chemokine ELC, fractalkine, macrophage inflammatory protein 1gamma MIP1 gamma, and stromal cell derived factor 1alpha SDF1 alpha.  Ref: 11290568 Am J Pathol, 2001
- ... monocytes macrophages readily produce chemokines such as RANTES, MIP 1alpha, MCP 1, MCP 3  [ **CCL7** ] , IP 10  [ **CXCR3** ]  and cytokines TNF alpha, IL 1beta, IL 6, IL 18 and IFN alpha beta.  Ref: 11163460 Vaccine, 2000
- The mRNAs encoding RANTES, MIP 1 alpha, MIP 1 beta, TCA3 I 309, IP 10  [ **CXCR3** ] , JE MCP 1, KC MGSA gro, and MARC  [ **CCL7** ]  MCP 3  [ **CCL7** ]  were induced in the spinal cord 1 2 days before clinical signs were apparent.  Ref: 7539012 J Neuroimmunol, 1995
- Under unstimulated conditions, no differences in the production of interleukin 8 IL 8, IFN inducible protein 10 IP 10  [ **CXCR3** ] , monokine induced by interferon gamma Mig, monocyte chemoattractant protein 1 MCP 1, and monocyte chemoattractant protein 3  [ **CCL7** ]  MCP 3  [ **CCL7** ]  were seen between untreated MS patients and controls.  Ref: 12020972 J Neuroimmunol, 2002
- Biopsies were stained for CD68, CD3, elastase, eotaxin, IP 10  [ **CXCR3** ] , MCP 1, MCP 3  [ **CCL7** ] , and IL 8 and analyzed by NIH Image analyzer.  Ref: 12827410 Int J Colorectal Dis< MedlineTA>Int J Colorectal Dis, 2002
- Influenza A virus infection results in the production of chemotactic RANTES, MIP 1 alpha, MCP 1, MCP 3  [ **CCL7** ] , and IP 10  [ **CXCR3** ] , pro inflammatory IL 1 beta, IL 6, IL 18, and TNF alpha, and antiviral IFN alpha beta cytokines.  Ref: 11325600 Cytokine Growth Factor Rev< MedlineTA>Cytokine Growth Factor Rev, 2002
- However, none of a series of radiolabelled chemokines IL 8, NAP 2, GRO alpha, PF4, IP10  [ **CXCR3** ] , MCP 1, MCP 2, MCP 3  [ **CCL7** ] , I 309, RANTES and MIP 1 alpha and other ligands C3a and leukotriene B4 bound to Jurkat transfectants that stably expressed either MDR15 or BLR1 mRNA.  Ref: 7639692 Biochem J, 1995
- The responses of cloned human NK cells ERNK57 to seven CC chemokines monocyte chemotactic protein 1 MCP 1, MCP 2, MCP 3  [ **CCL7** ] , RANTES regulated on activation, normal T cell expressed and secreted, macrophage inflammatory protein 1 alpha MIP 1 alpha, MIP 1 beta, and 1309 and two CXC chemokines IL 8 and IP 10  [ **CXCR3** ]  were studied.  Ref: 8598480 J Immunol, 1996
- Infection in the presence of protein synthesis inhibitor indicated that ongoing protein synthesis was required for influenza A virus induced expression of MCP 1, MCP 3  [ **CCL7** ] , and IP 10  [ **CXCR3** ]  genes.  Ref: 11022002 Virology, 2000
- Here we show that influenza A or Sendai virus infected human macrophages produce MIP 1alpha, MIP 1beta, RANTES, MCP 1, MCP 3  [ **CCL7** ] , MIP 3alpha, IP 10  [ **CXCR3** ] , and IL 8.  Ref: 11022002 Virology, 2000
- Several CC chemokines including eotaxin CCL 11, eotaxin 2 CCL 24, RANTES CCL 5, and monocyte chemotactic protein 3  [ **CCL7** ]  MCP 3  [ **CCL7** ] , CCL 7  [ **CCL7** ]  and 4 MCP 4, CCL 13 are potent eosinophil chemotactic and activating peptides acting through CC chemokine receptor 3  [ **CXCR3** ]  CCR3.  Ref: 10969084 J Biol Chem, 2000
- Of the chemokines tested MIP 1alpha, MIP 1beta, MCP 1, MCP 3  [ **CCL7** ] , RANTES, SDF 1, IL 8, IP 10  [ **CXCR3** ] , only MIP 1alpha, detected specifically on microglia, was expressed both constitutively and consistently.  Ref: 11746784 Glia, 2002
- In vitro addition of IFN beta1b to peripheral blood mononuclear cells PBMC cultures tended to decrease the production of IL 8, IP 10  [ **CXCR3** ] , Mig, MCP 1, and MCP 3  [ **CCL7** ] , but not of MIP 1alpha.  Ref: 12020972 J Neuroimmunol, 2002
